# Neural Responses to Musical Rhythm in Chinese Children With Reading Difficulties

**DOI:** 10.3389/fpsyg.2020.01013

**Published:** 2020-06-05

**Authors:** Chun-Han Chiang, Jarmo Hämäläinen, Weiyong Xu, Hsiao-Lan Wang

**Affiliations:** ^1^Department of Special Education, National Taiwan Normal University, Taipei, Taiwan; ^2^Department of Psychology, University of Jyväskylä, Jyväskylä, Finland

**Keywords:** reading difficulties, musical rhythm, magnetoencephalography (MEG), mismatch negativity (MMN), P3a

## Abstract

The perception of the musical rhythm has been suggested as one of the predicting factors for reading abilities. Several studies have demonstrated that children with reading difficulties (RD) show reduced neural sensitivity in musical rhythm perception. Despite this prior evidence, the association between music and reading in Chinese is still controversial. In the present study, we sought to answer the question of whether the musical rhythm perception of Chinese children with RD is intact or not, providing further clues on how reading and music might be interlinked across languages. Oddball paradigm was adapted for testing the difference of musical rhythm perception, including predictable and unpredictable omission, in elementary school children with RD and typically developing age-controlled children with magnetoencephalography (MEG). We used the cluster-based permutation tests to examine the statistical difference in neural responses. The event-related field (ERF) components, mismatch negativity (MMNm) and P3a(m), were elicited by the rhythmical patterns with omitted strong beats. Specifically, differential P3a(m) components were found smaller in children with RD when comparing the rhythmical patterns between predictable and unpredicted omission patterns. The results showed that brain responses to the omission in the strong beat of an unpredicted rhythmic pattern were significantly smaller in Chinese children with RD. This indicated that children with RD may be impaired in the auditory sensitivity of rhythmic beats. This also suggests that children with reading difficulties may have atypical neural representations of rhythm that could be one of the underlying factors in dysfluent reading development.

## Introduction

Basic auditory processing ability might be involved in both language development and music abilities across cultures (Hämäläinen et al., 2009; Goswami et al., 2011; Flaugnacco et al., 2014). Some previous studies have directly examined the relationship between music and reading development (Anvari et al., 2002; David et al., 2007). Interestingly, children with reading difficulties show deficits in temporal processing in both reading and music domains. Especially, rhythm ability was found as a strong predictor of reading abilities in 53 children in a 5-year longitudinal study (David et al., 2007). The report indicated that rhythm ability was not only significantly correlated with phonological awareness and naming speed but also predicted unique variance in pseudo-word reading ability 5 years later (David et al., 2007).

Goswami et al. (2011) have assessed the rhythm perception and speech prosody perception in individuals with dyslexia and showed dyslexic persons had difficulties in processing prosodic cues, such as the amplitude onset or rise time of sounds (Goswami et al., 2010, 2011). Therefore, poor sensitivity for auditory cues might lead to difficulties in the perception of rhythmic structure of speech, which also could affect the development of phonological awareness for reading skill development. For example, 10-year-old children with dyslexia were significantly poorer in musical metrical perception than age-matched children with typical reading abilities, but they performed equally well as 8-year-old children who were matched by reading level (Huss et al., 2011). In a follow-up study, the same musical tasks were re-administered after 1 year. Those dyslexic children did significantly worse in the musical perception tasks than reading level matched children (Goswami et al., 2013). Also, Huss et al. (2011) showed that the music metrical sensitivity predicted phonological awareness and reading abilities and the metrical performance explained over 60% of the variance in reading along with IQ and age; the music perception (like rhythm perception) even showed stronger associations with reading than the abilities of phonological awareness.

From a neurobiological perspective, speech and music perception are two processes using largely overlapping anatomical brain networks. Both auditory cues of language and music are processed through the same pathway (Kraus and Chandrasekaran, 2010; Patel, 2011). Specifically, rhythm is an essential component to understand speech and developmental studies suggest that infants are not only sensitive to a regular pulse, but also to meter and rhythm (Winkler et al., 2009; Suppanen et al., 2019). Difficulties in decoding auditory signals could affect reading development in children through speech stress awareness (Goswami, 2011). The syllable stress in speech is reminiscent of alternating the clear accent in music, such as the strong beat. There are some studies examining the processing of rhythm cues in both infants and adults. Winkler et al. (2009) examined the processing of beats in sound streams in 14 healthy newborn infants between 37 and 40 weeks gestational age. For measuring the electrical brain mismatch negativity (MMN) to sounds, they presented sound sequences based on a repetitive rhythmic pattern of traditional western music that randomly omitted sounds in strong or weak beats while the neonates were sleeping. The results showed significantly different electrical brain responses to early (200 ms) negative and late (438 ms) positive waveforms between standard (rhythmic pattern) and deviant (rhythmic pattern with omissions at the strong beat) stimuli. They concluded the newborn babies detected the violation of a beat in a rhythmic sound pattern. Therefore, the capability to beat perception in rhythm sound sequence seems to be innate. Using a similar event-related potential (ERP) experimental paradigm as Winkler et al. (2009), Bouwer et al. (2014) examined the beat processing with strong accents for adult musicians and non-musicians in an un-attentive condition. Although an MMN was elicited by all of the omissions for both musicians and non-musicians, they found that the omission in the strong beat position elicited larger amplitude MMN response than the omission in the weak metrical position but there was no significant effect of musical expertise for detecting the metrically simple rhythms. Furthermore, another study that used similar experimental stimuli also indicated that the beat rhythms of real music could lead to stronger effects (earlier and larger amplitude) of metrically rhythmic processing even in healthy adults without any long-term musical training (Ladinig et al., 2009). However, Geiser et al. (2009) compared the meter, rhythm, and pitch perception between musicians and non-musicians in attended and unattended conditions. In behavioral measurements, un-trained participants were significantly poorer than well-trained musicians in metrical abilities, but they were as good as musicians in rhythm perception. As expected, the electroencephalography (EEG) results showed that the violation of rhythm, as well as meter, elicited an early negative amplitude (MMN-like component) compared to standard stimulus in the attended processing condition; meanwhile, in the unattended condition, only rhythmic deviants elicited the negative deflection in both groups. To date, there is a lack of studies utilizing real music rhythm to measure the rhythm induction for children with dyslexia. Even though the effects of musical training has been examined in individuals with reading problems (Cancer et al., 2019), but the rhythm induction of brain response in real musical beats for dyslexic children is still not investigated.

In addition to MMN, P3a component was also elicited by pitch, vowel, intensity, and rhythm deviations in sound streams (Lelo-de-Larrea-Mancera et al., 2017; Linnavalli et al., 2018). Especially, musicians showed a larger P3a waveform to deviant acoustic stimulus than non-musicians (Donchin and Coles, 1988; Putkinen et al., 2019). The P3a component, a positive amplitude around 300 ms, is originally assumed as a representation of involuntary attention capture triggered by salient deviant sounds (Escera et al., 1998; Escera and Corral, 2007). However, it is not conclusively interpreted as any neural or cognitive representation but most explanations are related to context updating (Luck, 1963; Squires et al., 1975; Donchin, 1981). Some studies showed that musicians have more sensitivity to suprasegmental cues in speech (native or non-native) and even a small change in pitch could induce significant brain responses in musicians; also, the P3 component, induced by speech, showed stronger responses in music-trained participants than those who have no music training (Schön et al., 2004; Marques et al., 2007). Cason and Schön (2012) designed an oddball paradigm that presented music-like patterns followed by stress-matched syllables. When the stress of syllables did not match the predictive rhythm, stronger and longer-lasting P3 responses were observed. The mechanism of attentional orienting (reflected by the P3a response) should enhance the processing of phonological awareness. Once the prosody of speech did not synchronize with predictively rhythmic beats, it would consume more cognitive abilities for responses, and vice versa (Cason and Schön, 2012).

The P3a component in dyslexia has not been studied extensively. Recently, Putkinen et al. (2019) investigated the cortical responses of MMN and P3a in children and adolescents with/without musical training. The musically-trained participants responded to infrequent deviant sounds and showed stronger P3a components than non-trained with significant correlation with reading abilities (pseudo-word test). The study was in line with the assumption that P3a probably was strongly related to reading abilities and musical training could enhance the neural-level discrimination of infrequent deviant sounds (Putkinen et al., 2019). In other studies, the P3a component showed no difference to violation of musical patterns between musicians and non-musicians in un-attended experimental designs, since the brain would probably have innate neural mechanism to process such unexpected sounds without need for eliciting attention switching to the sounds (Bouwer et al., 2014, 2016).

Beats in music are an important component of rhythm. Beat induction, the regular detection in an auditory signal, is suggested to be fundamental and inherent in human ability without learning, because the MMN response of newborn infants is elicited by the deviant stimuli from expectation, especially on the beat perception task (Winkler et al., 2009). Existing literature suggests that the brain responses of dyslexic children and adults show atypical processing of rhythmic beats and also deficits in speech prosody (Thomson et al., 2006; Thomson and Goswami, 2008; Tierney and Kraus, 2013a, b; Woodruff Carr et al., 2014). Especially, Chinese children with dyslexia showed poorer rhythm imitation abilities than children with typical development; meanwhile, a significant correlation was found between rhythmic abilities and phonological awareness (Lee et al., 2015). From above studies, we expect detection of deviations in beats in sound series to be associated with readings skills, particularly to lower sensitivity of beat perception in individuals with reading difficulties (RD) than typical developmental controls. Specifically, we anticipate the MMN and P3a responses to be larger for typically developing children than children with RD for the omission deviants in strong positions.

Here we implemented a passive oddball paradigm including standard sounds with musically rhythmic beats and deviant sounds with omission beats. The purpose of this study was to explore the difference of neural responses in musical rhythm between typically developing children and children with RD. The assumption was based on the following behavioral and neural studies: children and adults with dyslexia had deficits in processing of rhythmic patterns both in speech and music (Goswami et al., 2002; Goswami, 2011; Huss et al., 2011); after musically based training/intervention, the brain responses, phonological abilities, and reading skills were enhanced in children (Bhide et al., 2013; Flaugnacco et al., 2015; Serrallach et al., 2016; Putkinen et al., 2019); the neural rhythmic entrainment of dyslexics was not as well synchronized as typical groups (Colling et al., 2017). Therefore, we anticipated the MMN and P3a to musical rhythm in dyslexic children to show a different pattern compared with their peers with typical development.

## Materials and Methods

### Participants

Thirty-two elementary school students from grade 3rd to grade 4th, participated in this study. Sixteen were diagnosed with reading difficulties (13 males) by local Committee Responsible for Identification and Placement of gifted and disabled students. The participants with reading difficulties (referred to as RD group) had received special education in resource rooms and had no diagnosis of ADHD. Sixteen participants (11 males) were typically developing children acting as age-matched control group (referred to as AG group). None of the participants reported a history of neurological or hearing problems.

All participants and their parents were given written informed consent before the study. The study was approved by the Ethics Committee of the National Taiwan Normal University. All the comparisons related to reading-related skills between RD and AG group are shown in Table 1. The AG had higher scores than RD in verbal (Similarity) abilities, tone awareness, onset-rime awareness, Chinese character recognition, and reading comprehension, but non-verbal abilities (Matrix reasoning) showed no significant group difference.

**TABLE 1:** Summary of reading related measures and results of independent samples *t*-test between the age-matched control group (AG) and group with reading difficulties (RD).

**Variables**	**Group**	***N***	**Mean**	***SD***	***t***	***df***	**Sig. (2-tailed)**
Age (month)	AG	16	115.5	8.07	0.92	30	0.927
	RD	16	115.25	7.22			
Similarity	AG	16	12.44	2.12	4.61	30	0.000
	RD	16	8.56	2.61			
Matrix	AG	16	9.37	3.07	0.919	30	0.336
	RD	16	8.50	2.25			
PPVT (%)	AG	16	75.88	10.45	5.72	30	0.000
	RD	16	47.56	16.80			
TA (raw)	AG	16	15.63	2.39	3.55	22.93^#^	0.002
	RD	16	11.13	4.47			
ORA (raw)	AG	15	17.27	3.39	4.05	29	0.000
	RD	16	11.44	4.50			
Character recognition (raw)	AG	16	95.13	18.85	4.38	30	0.000
	RD	16	63.50	21.92			
Reading comprehension (raw)	AG	16	21.75	3.53	8.67	30	0.000
	RD	16	9.88	4.19			

**AG, age-matched control group; RD, Reading-difficulties group; #, Levene’s test for equality of variances showed unequal variances.**

All participants were administered the following behavioral measurements: (1) The Chinese Peabody Picture Vocabulary Test -Revised (PPVT-R; Lu and Liu, 1998) was translated into Chinese Mandarin. This assessment is frequently used for children aged between 3 and 12 years old. Children are shown four numbered pictures and are read a word by the administrator. The child is then asked to point to the picture or to say the number of the picture that corresponded to the word spoken by the administrator; (2) Phonological Awareness Composite included tone awareness (TA) and Onset-Rime Awareness (ORA). TA test included four practice trials and 24 testing trials. Each trial contained four different syllables (e.g., /xau1/, /pei1/, /p^h^a3/, /liǝ1/). Among the 24 testing trials, 12 were nonsense syllables. Children were required to select the syllable with the odd tone they heard from the administrator (e.g., /p^h^a3/). One point was given if they answered correctly. ORA test was similar to the tone awareness test, children were asked to choose the odd syllable which had a different initial or final sound from the other three syllables. For example, /pǝn4/ is the answer in the item /ni4/, /pǝn4/, /nau4/ because it presents a different initial sound. There were four practice trials and 30 testing trials. Within the testing trials, half of the trials were initial oddity tests and the rest were final ones. Among the 30 testing trials, 15 of them used nonsense syllables; (3) Graded Chinese Character Recognition (Huang, 2001), the child is asked to read a series of 200 characters aloud that got progressively lower in printed word frequency. The accuracy of children’s responses was reported. For this timed test, its split-half test reliability coefficient was 0.99; (4) Reading Comprehension Screening: This measure was designed by Ko and Chan (2006) to examine reading comprehension of 2nd to 6th graders. There are two sections included: paragraph review and text comprehension. Children are asked to name the topics, to find out the synonyms, to reason and interpret the writing. The accuracy of children’s responses was reported. The test-retest reliability coefficient of this test was 0.87.

### Stimuli and Experiment Design

For studying the effects of the rhythm, we adopted an oddball paradigm, similar to Winkler et al. (2009). The experiment used a non-stop music stream composed of five different rhythmic patterns consisting of three different sounds, drum, snare, and hi-hat. The music sounds were created by Logic Pro X (Apple Inc.). The intensity of the drum was the largest and the smallest intensity was the hi-hat. Then, the strongest (drum) and softest (hi-hat) sounds were assumed as the metrically strongest and weakest beats, respectively. The assumption was in line with the way of Western music that the drum was in the position of the downbeat and the hi-hat was used for subdivision at the metrically weakest level.

**FIGURE 1:**
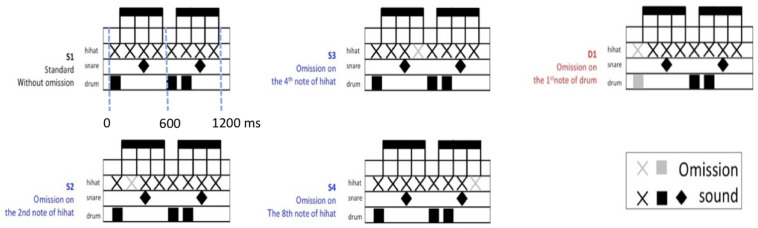
Schematic patterns of musical rhythm stimuli.

Four rhythmic patterns were used as a standard pattern (S1) and three sub-standard patterns (S2, S3, and S4) with one used as deviant rhythmic pattern (D1) (see Figure 1). The standard pattern (S1) consisted of eight consecutive sounds, with an inter-onset interval of 150 ms and a total length of 1,200 ms. The inter-onset intervals are 50, 100, and 150 ms for hi-hat, bass drum, and snare drum, respectively. The sub-standard rhythmic patterns (S2, S3, and S4) with omission in metrically weak position (sound of hi-hat) were in contrast to the deviant pattern (D1) with omission in the first metrically strong position (sound of drum). Though S2, S3, and S4 had different omission in each weak position, the rhythmic patterns were still perfect meters. Therefore, those rhythmic patterns, S2, S3, and S4, were categorized as sub-standard beats instead of omitted strong beats or deviant patterns.

Participants were seated in the magnetoencephalography (MEG) machine (Elekta Neuromag^®^) in a soundproof and magnetically shielded room. They watched a self-selected movie without subtitles and sounds on a projection screen and listened to the auditory rhythmic stimuli in an unattended condition. Participants were instructed to focus on the quiet movie and not to pay attention to the stimuli. The experimental session includes 3 test blocks and one deviant-control (DC) block presented by Presentation software. Three test blocks comprise 276 standard (S1–S4) and 30 deviant (D1) patterns. Additional 5 S1 were added to the beginning of each test block, but DC block consisted of 300 D1 patterns. Every test block of stimuli was followed by a break of few minutes. In the test blocks, S1–S4 appeared with equal 22.5% probability and the D1 pattern appeared with 10% probability. The order of the 5 patterns is pseudo-randomized. Two constraints are for every block: (1) At least 3 standard patterns between successive D1 patterns; (2) Deviant pattern can’t precede standard pattern S4 because it would make two successive gaps. Patterns in the sequence were delivered without breaks. The loudness of the sounds was normalized so that all stimuli, including the strong beats, had the same loudness when exporting to mp3 files.

### MEG Data Acquisition, Preprocessing, and Analysis

We used a 306-channels MEG system (Elekta-Neuromag, Helsinki, Finland) located at National Taiwan University to record continuous neural responses, both 102 sensors of magnetometers and 204 gradiometer sensors (combined into 102 signals using vector sum) and were used for data analysis by the BESA 6.0 program (high-pass filtering: 0.5 Hz; low-pass filtering: 40 Hz; sampling rate: 1000 Hz). To reduce the noise from the head movements, we used signal space separation method (Taulu and Kajola, 2005) to individually correct MEG recording with head position indicator (HPI) coils in the nasion and two (left and right) preacuricular anatomical landmarks. The artifacts including heart beats and eye movements were linearly reduced by using ICA (Independent Component Analysis) and the waveforms were visually inspected and any large artifacts were manually rejected. The average rate of rejected epochs from S1, D1, and DC were 47.50, 32.50, and 38.65%, respectively.

The non-parametric permutation tests were used with magnetometer and gradiometer channels and time-point clustering, to examine statistical differences in the responses within and between groups by BESA Statistics 2.0. The number of permutations testing was set to 1,000 for each comparison at alpha of 0.05 and the neighbor distance of channel cluster was set to 4 cm for within- and between-group comparison (Maris and Oostenveld, 2007). For comparing the MMNm (100–200 ms), P3am (250–350), and Reorientation negativity (RONm, 400–600 ms), the ERFs in the interval 100–600 ms were analyzed.

All responses from -200 to 600 ms were averaged for the S1, D1, and DC patterns. A baseline of -200–0 ms before standard and omission stimuli were used. Amplitudes were analyzed by using non-parametric permutation tests to examine group differences of MMNm, P3am, and RONm. We focused on the processing of the strong position of the omitted rhythmic beats which was analog to the stress of speech that was assumed as deficient in dyslexics (Goswami, 2011; Huss et al., 2011). Instead of the omitted sound in the metrically weak position (S2, S3, and S4), here we only compared the deviant pattern with omission in the first metrically strong position (D1 and DC) for comparing with standard stimuli (S1).

## Results

### Within Group Comparison in Control Group

**FIGURE 2:**
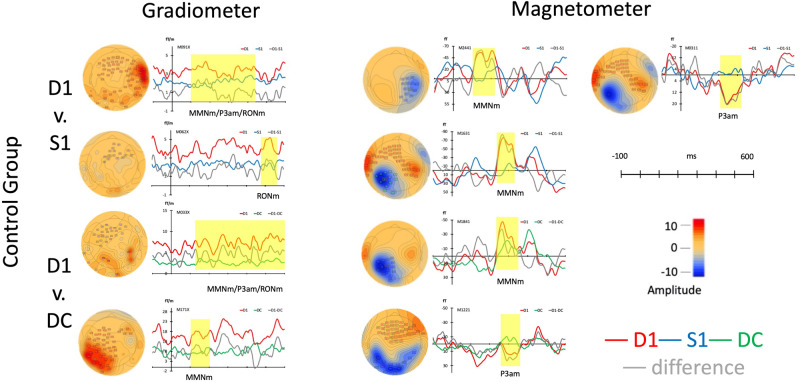
Average responses from gradiometers and magnetometers and topographies from control group in each permutation test (within subjects).

In control group (*N* = 16), the cluster-based permutation *t* test was used for comparing three stimuli including S1, D1, and DC in separate types of channels. In gradiometer channels (the left panel of Figure 2), the pairwise comparison of permutation *t* tests were as following: Comparing D1 deviant to standard stimulus (S1), there were two clusters in different time points. the D1 pattern showed a distinct waveform peak at the time point, 100–447 ms (*p* < 0.001, Mean: D1 = 11.06; S1 = 6.22) and 471–560 ms (*p* < 0.05, Mean: D1 = 5.47; S1 = 3.55). The topographies and time windows were suggesting the source as MMNm, P3am, and RONm. Also, comparing D1to DC, there were two clusters with a peak difference at 127–599 (*p* < 0.001, Mean: D1 = 9.12; DC = 5.19) ms and 100–205 ms (*p* < 0.01, Mean: D1 = 6.53; DC = 3.71) and those topographies and amplitudes suggesting the components as MMNm, P3am, and RONm.

In the magnetometer channels as seen in the right panel and upper part of Figure 2, there were three clusters when comparing D1 to S1. The waveforms of D1 had stronger responses at 219–330 ms (*p* < 0.001, Mean: D1 = 17.41; S1 = -3.17), 100–224 ms (*p* < 0.01, Mean: D1 = -35.87; S1 = -1.54), and 239–331 ms (*p* < 0.05, Mean: D1 = -47.17; S1 = -1.53) than S1. The two clusters were suggested as MMNm (100–224 ms and 239–331 ms) and the response represented P3am showed around 300 ms (219–330 ms). When comparing D1 to DC, two clusters were found at the time windows including 274–356 ms (*p* < 0.01, Mean: D1 = 12.26; DC = -3.30) and 23–376 ms (*p* < 0.01, Mean: D1 = -31.33; DC = -2.79) in the right panel and lower part of Figure 2. One response was suggesting as MMNm (237–376 ms) and the other one was resembling P3am.

In the control group, the distinctive amplitudes of MMNm, P3a, and RONm were found when the ERFs were elicited by the deviant musical rhythm with omission in the strong beat. The significant responses of all responses can be seen from the different waveforms and topographies in Figure 2.

### Within Group Comparison in Group With Reading Difficulties

**FIGURE 3:**
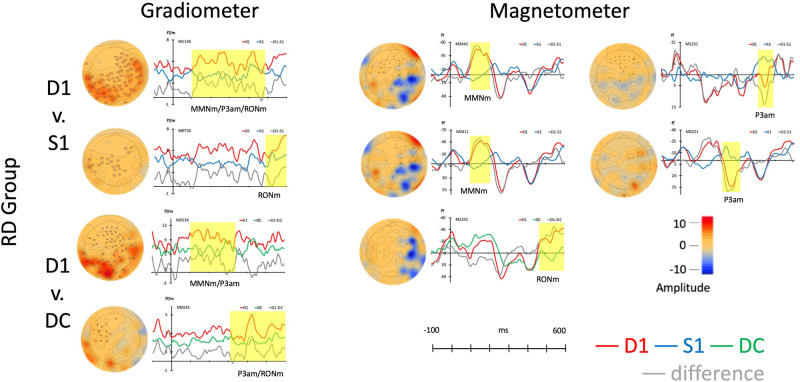
Average responses from gradiometers and magnetometers and topographies from RD group in each permutation test (within subjects).

The same permutation test that used for the control group was adapted for RD group. In the gradiometer channels (the left panel of Figure 3), the results of D1 to S1 revealed two clusters including a significant response in the time windows at 100–489 ms (*p* < 0.001, Mean: D1 = 8.98; S1 = 4.96) and 486–599 ms (*p* < 0.05, Mean: D1 = 6.99; S1 = 4.60). The components across 100–599 ms resembled MMNm, P3am, and RONm. While comparing D1 to DC, the D1 had two clusters in the time windows at 302–599 ms (*p* < 0.001, Mean: D1 = 7.82; DC = 4.74) and at 100–337 ms (*p* < 0.01, Mean: D1 = 9.76; DC = 5.77). Those waveforms also resembled MMNm, P3am, and RONm.

In the magnetometer channels from the right panel of Figure 3, only one strong response was suggested as RONm at the time window from 466 to 599 ms (*p* < 0.05, Mean: D1 = -23.36; DC = -0.83) when comparing D1 to DC. Although the MMNm (time: 100–208 ms, *p* < 0.05, Mean: D1 = 16.75; S1 = -2.27; time; 107–215 ms, *p* < 0.05, Mean: D1 = -33.56; S1 = 3.06) and P3am (time: 221–321 ms, *p* < 0.05, Mean: D1 = 25.70; S1 = -5.61; time: 417–495 ms, *p* < 0.05, Mean: D15.25; S1 = -3.88) components were found when comparing S1 to D1, there was no distinctive RONm amplitudes when comparing D1 to S1.

Children with reading difficulties also showed sensitivity to the musical rhythm that elicited three different components, MMNm, P3am, and RONm, in both gradiometer and magnetometer channels. All responses and topographies of RD group are in Figure 3.

### Comparison Between the Groups

**FIGURE 4:**
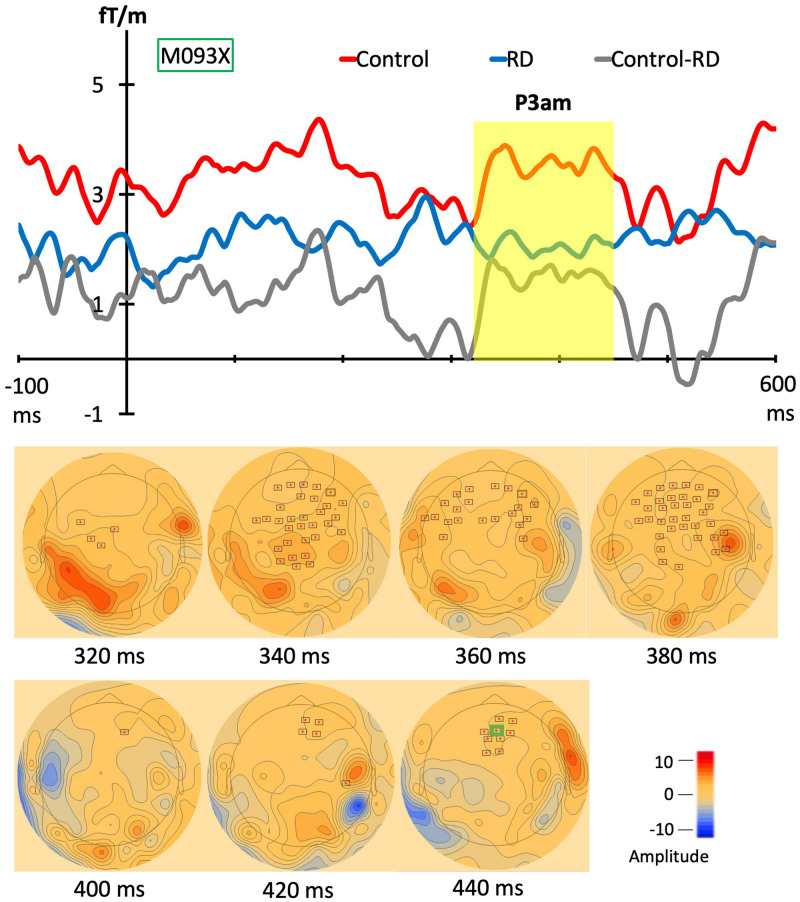
Grand evoked responses from gradiometers and topographies. The topographies in lower panel shows the significant group difference in combined gradiometer channels (red dots with black frames). In the upper panel, the averaged magnetic evoked responses from control group (red line) and RD group (blue line). The gray line represents the responses of grand waveforms of controls minus RD group and the yellow block shows the time windows of the significantly different responses between groups. The topographies represent the clusters and most of them are in the frontal and parietal lobe.

To explore the statistically significant differences for each stimulus between control and RD group, the permutation *t* test (Number of permutations: 1000; neighbor distance: 4 cm) with spatiotemporal clustering was adapted for both gradiometer and magnetometer channels. In the D1 stimulus from Figure 4, one cluster between two groups was found at 315–455 ms (*p* = 0.033, Mean: control = 5.02, RD = 2.89) in gradiometer channels. However, no statistically significant effects were found when comparing DC stimulus between two groups as well as standard stimulus (S1) in both different types of channels. Above, only P3am component was revealed but no other significant difference was found. The continuing topographies and amplitudes from gradiometer channel (M093X) of each group are in Figure 4.

## Discussion

In this MEG study with MMN-paradigm, musical rhythm was used to study the auditory perception of children with reading difficulties compared to children with typical development. The stronger ERFs were found to rhythmic omissions in children with typical development than children with reading difficulties. Comparing the rhythmic violation to the same pattern with regular omissions in control group, the MMNm, P3am, and RONm showed strong peaks from 100 to 600 ms. The findings are consistent with recent studies of rhythmic omissions that people can elicit the discriminative MMN response to the irregularly omitted strong beats (Winkler et al., 2009; Bouwer et al., 2014), the P3a component to deviant beats in the unattended condition (Bouwer et al., 2016), and the reorientation negativity switching the attention back to the original stimulus (Horváth et al., 2008). These results demonstrate that the omitted downbeat elicits stronger brain responses and the beat perception is a fundamental ability for the children with typical development (Geiser et al., 2009).

However, we also found the strong components, MMNm, P3am, and RONm, were elicited in children with reading difficulties when comparing the pattern with omissions to the pattern with regular rhythm. Interestingly, an obvious component, P3am, was showing higher response to the omitted strong beat in normal children than children with reading difficulties. The findings show that children with reading difficulties are impaired in the auditory perception of rhythmic beat, especially in the strong beats. In Goswami’s study, the rhythm perception and speech prosodic abilities of individuals with dyslexia were impaired in prosodic cues, such as rise time (Goswami et al., 2010, 2011). Also, Hämäläinen et al. (2012) had also shown adults with dyslexia had difficulties to be sensitive to low amplitude modulation stimuli. The deficits in sensitivity to auditory cues could lead to difficulties in the rhythmic structure of speech, which also could affect the development of reading abilities. Few studies have found that auditory perception has the relationship with rhythmical and prosodic tasks (for review, Goswami, 2011); meanwhile, the impairment of rhythm production (tapping with a metronome) was found both in children with dyslexia (Thomson and Goswami, 2008) and dyslexic adults (Thomson et al., 2006). Those difficulties in reproducing simple rhythms by tapping were also associated with the speech rhythm, phonological processing, and reading (Wolff, 2002; Thomson et al., 2006). Above studies indicated dyslexics might have impairment in rhythm perception and even production which is related to the reading abilities.

Previous studies showed P3a component, in response to to novel or unexpected auditory stimuli, might be related to context updating (Squires et al., 1975; Donchin, 1981), working memory (Breznitz and Meyler, 2003), or attention (Yang et al., 2015). Especially, people with P3a deficit demonstrated problems in cognition and behavior (Ford et al., 2018). In the current study, we found that the smaller P3a component in the RD group indicates children with reading difficulties were less sensitive to the deviant rhythmic patterns than those children without reading problems. The results of this study are similar to previous research in children and adolescents (Maciejewska et al., 2013). It suggests that even the preliminary detection of violation didn’t reflect group differences by the MMN component, but a following auditory processing stage, P3a, probably represented stronger relationship in the children without reading problems than in the ones with reading disabilities. The results also are in line with previous studies showed children with musical training have more sensitive P3a components than those without musical training (Donchin and Coles, 1988; Vuust et al., 2009; Putkinen et al., 2019). For instance, the amplitude of P3a showed significant correlation with pseudo-word reading scores after partialing out the effect of age and group membership (Putkinen et al., 2019). The correlation between neural auditory stimuli processing and reading abilities, like pseudo-word reading in previous study (Putkinen et al., 2019) provides more evidence to support that the P3a component might be a potential precursor for children with reading problems (Corbera et al., 2006). Also, the musical training could improve the neural responses for children to efficiently process not only rhythms but also speech and reading abilities (Bhide et al., 2013; Bonacina et al., 2015; Flaugnacco et al., 2015). Also, P3 was related to general factors about cognition, awareness, and attention switch (Schulte-Körne et al., 1999; Maciejewska et al., 2013); meanwhile, P3 could be an index for evaluating the error and it represented the relationship between tapping synchronization and auditory-motor network (Kamiyama and Okanoya, 2014). The P3 component which responded to speech sound processing had already developed in young children and a study showed there was no difference between typically developing children and adolescent; but it was still undeveloped in dyslexic children and the P3 component was significantly larger in dyslexic adolescents than in young dyslexic children (Maciejewska et al., 2013). Therefore, the differences in the P3 component could lead to difficulties to process speech related to reading development. Thus, we favor the idea that P3a is related to temporal predictability easing the cognitive load during speech processing (Cason and Schön, 2012) and the P3a may rather reflect possibly higher-level event-detection process than attention switching itself (Horváth et al., 2008). For instance, Cason and Schön (2012) had utilized musical rhythm to enhance phonological processing of spoken words. When the rhythmic expectations were not met with the prosodic feature of speech, the P3 component was larger than rhythmic expectations matched prosody and predicted the efficiency of phonological processing.

However, there was no significant group difference of MMN component in contrast with previous studies showing stronger MMN responses to speech and pure tones in controls than group with reading difficulties (Schulte-Körne et al., 1999; Maciejewska et al., 2013). It remains an open question whether the deficits found in rhythm perception are related to an underlying deficit in Chinese dyslexics. Probably, the limitation of the small sample size, different stimuli, experimental design, or different language had an effect on the results (Leppänen et al., 2019). It is necessary to conduct more research on individuals with reading difficulties to investigate the extent of musical rhythm deficit in neural processing as well as other abilities that have been shown to correlate with phonological awareness and reading skills.

This study highlights the relationship between reading and musical rhythm processing in Chinese children with reading difficulties. We have shown the basic rhythm perception doesn’t require attention for the elicitation of MMNm and P3a(m) responses to omissions in acoustically rhythmic stimuli in normal children and this conclusion is in line with the previous adult and newborn studies. Furthermore, the smaller ERF responses, especially P3a(m), in children with reading difficulties was found in rhythm processing. This indicates that children with reading difficulties have abnormal neural representations of rhythm.

## Data Availability Statement

The datasets generated for this study are available on request to the corresponding author.

## Ethics Statement

The studies involving human participants were reviewed and approved by National Taiwan Normal University. Written informed consent to participate in this study was provided by the participants’ legal guardian/next of kin.

## Author Contributions

C-HC developed and tested the MEG procedure under the supervision by H-LW and JH. C-HC, JH, and WX analyzed the MEG data. C-HC drafted the manuscript. H-LW, JH, and WX revised the manuscript. All authors contributed to the discussion and agreed with the final version of the manuscript for submission.

## Conflict of Interest

The authors declare that the research was conducted in the absence of any commercial or financial relationships that could be construed as a potential conflict of interest.
